# Angiostatic vaccines, an underestimated approach of cancer therapy

**DOI:** 10.18632/oncotarget.2791

**Published:** 2014-11-17

**Authors:** Arjan W. Griffioen

**Affiliations:** Angiogenesis Laboratory, Department of Medical Oncology, VU University Medical Center, Amsterdam, The Netherlands

The paper by Femel et al. [1] demonstrates the success of a vaccine against the extra domain A of fibronectin, an embryonic gene product that is re-expressed during neoformation of blood vessels. This vaccine is able to significantly suppress both primary tumor growth, as well as metastasis formation in a spontaneous breast cancer mouse model. This paper hallmarks the progress and impact of this promising strategy of angiostatic cancer treatment.

While 2013 has been selected by Science's editors as the year of cancer immunotherapy for the positive clinical results on two immune checkpoint inhibitors, the field of cancer vaccination is also picking up slowly after a 30-year period of gains and losses. The attractiveness of cancer vaccination is clearly the harnessing of the immune system, which we know is capable of recognizing and destroying tumors and even able to cure patients in rare cases. Cancer vaccines are biological response modifiers aiming at redirecting or focusing the body's immune response to destroy the tumor. A specific and underestimated class of these vaccines is designed to recognize the tumor vasculature.

Inhibition of angiogenesis has long been considered an attractive adjuvant option for the treatment of cancer. The early detection of vascular endothelial cell growth factor as a major regulator of angiogenesis and the development of its inhibitor bevacizumab has focused the field's attention mainly towards this and other growth factor signaling pathways. But it seems that a ceiling has been reached with these drugs in the prolongation of patient survival. It is therefore suggested that the direct targeting of tumor endothelial cells may be a better option. Since it is already known that inhibition of angiogenesis has a positive impact on anti-tumor immunity [2], using the immune system to target the tumor vasculature seems to combine the best of two fields. A vaccination approach to angiostatic cancer therapy is likely to serve this goal. Very early support for the promising nature of this field was provided by Wei et al., who showed that immunization with paraformaldehyde fixed xenogeneic whole cell preparations of purified human endothelial cells (either HUVECs or glomerular endothelial cells) inhibited tumor growth in both prophylactic and therapeutic settings [3]. Another early study showed that a DNA vaccine targeted towards the endothelial migration associated molecule angiomotin, induced a marked inhibition of tumor growth in immunized mice [4].

A major requirement for the success of a vaccination approach against the vasculature is the availability of highly specific targets. Several studies have reported on increasingly specific markers of the tumor vasculature [5], allowing the induction of specific immunity. The study by Femel et al. used the approach of conjugating the self-antigen to a strong bacterial antigen, a strategy that was also successful in a previous study by the same group on a vaccine against the extra domain B of fibronectin [6]. Injection of this conjugate together with an appropriate adjuvant resulted in the induction of a strong antibody response against the self-antigen. This strategy seems now very successful as a similar approach was recently published by Zhuang et al. on vaccination against the angiogenesis marker robo4 [7]. Major advantages of such vaccination approaches are (i) that a polyclonal antibody response is induced, which has better antigen neutralizing capacities; (ii) that vaccines do not require to be frequently administered, as is the case with monoclonal antibodies and small molecule drugs, and (iii) that vaccination provides an extremely cost effective strategy, which can be up to a hundred times less expensive than a monoclonal antibody therapy, due to the low amount of protein required for immunization.

The papers by Femel and Zhuang highlight the impact of this exciting field of research, which is anticipated to confirm similar efficacy in clinical trials in the near future.
